# Hidden in Plain Sight—An Unusual Case of Acute-onset Persistent Dyspnea

**DOI:** 10.19102/icrm.2023.14071

**Published:** 2023-07-15

**Authors:** Attila Roka, Hussam Abuissa, Kishan K. Srikanth

**Affiliations:** ^1^Division of Cardiovascular Medicine, Creighton University, Omaha, NE, USA

**Keywords:** Atrial fibrillation, electrocardiogram, pacemaker syndrome

## Abstract

Pacemaker (PM) syndrome is an uncommon complication after PM or defibrillator implant in patients with long-standing persistent atrial fibrillation. We present a case where an unexpected and unrecognized improvement in a comorbid condition paradoxically led to worsened symptoms, ie, acute-onset persistent dyspnea, in a patient with a single-chamber implantable cardiac defibrillator. A careful review of clinical data led to diagnosis and successful treatment.

## Case presentation

An 84-year-old man presented to the electrophysiology clinic with persistent dyspnea and decreased exercise tolerance, with a sudden onset 2 months prior.

**Figure 1:**
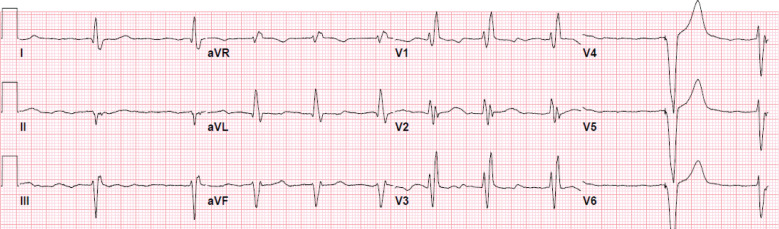
Electrocardiogram obtained after VVI implantable cardioverter-defibrillator placement; the pacing rate was set at 40 ppm. Note the atrial fibrillation with right bundle branch block and left anterior fascicular block.

His medical history included ischemic cardiomyopathy with a left ventricular ejection fraction (LVEF) of 35% and severe aortic valve stenosis, for which he underwent 3-vessel coronary bypass graft surgery and a Carpentier–Edwards bioprosthetic aortic valve replacement procedure 8 years ago. Six years after his operation, minimally symptomatic persistent atrial fibrillation (AF) was noted, the LVEF had improved to 65%, and rate control and anticoagulation were pursued. A year later, he had an episode of syncope and several episodes of presyncope. Sustained ventricular tachycardia was noted while he was monitored on hospital telemetry. The LVEF was 35%–40%. Ischemic re-evaluation confirmed 1 occluded graft due to competitive flow and 2 functional grafts; no further revascularization was pursued. The AF persisted with controlled heart rate, and the QRS duration was 148 ms with right bundle branch block and left anterior fascicular block. Amiodarone was started and a single-chamber implantable cardioverter-defibrillator (ICD) was implanted (Visia AF VVI ICD; Medtronic, Minneapolis, MN, USA), with the following device settings: VVI pacing at 40 ppm, ventricular tachycardia detection at 162 bpm, and ventricular fibrillation detection at 231 bpm. Cardiac medications on discharge consisted of amiodarone, metoprolol succinate, apixaban, and spironolactone. Follow-up electrocardiography (ECG) showed AF with controlled heart rate and demand ventricular pacing **(Figure 1)**.

**Figure 2:**
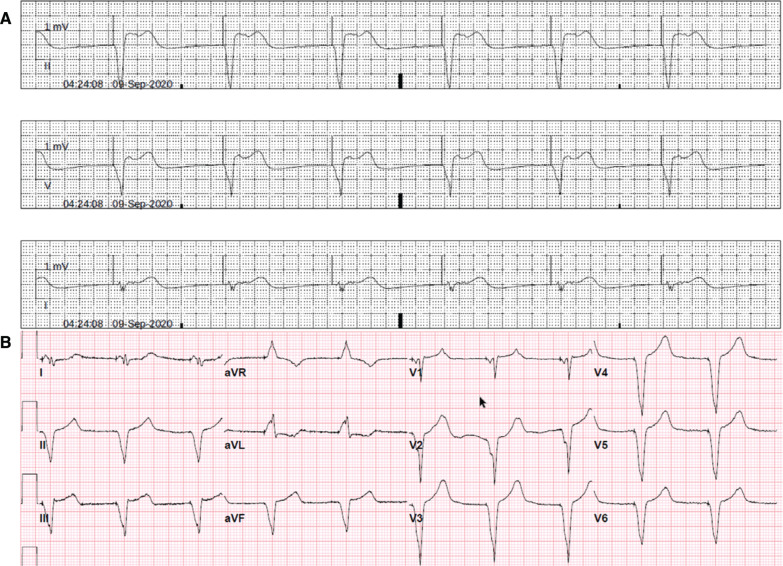
Electrocardiograms obtained during the patient’s emergency room visit. **A:** Telemetry tracings on presentation. Ventricular pacing at 40 bpm. **B:** Ventricular pacing at 60 ppm. Notching in the T-waves suggests the existence of hidden P-waves, with constant ventriculoatrial delay.

One year later, he noticed sudden-onset persistent fatigue and dyspnea and was evaluated in an emergency room a few days after the onset of symptoms. Bradycardia was noted at 40 bpm **(Figure 2A)**. Acute coronary syndrome and pulmonary embolism were ruled out with biomarkers, and normal cell counts were noted. A chest radiograph showed no active pulmonary pathology. An echocardiogram showed normal bioprosthetic aortic valve function, normal pulmonary arterial systolic pressure, and an LVEF of 60%. The ICD was interrogated, and normal lead parameters were noted, with sudden bradycardia prior to the onset of symptoms. The pacing rate was increased to 60 ppm **(Figure 2B)**, with minimal improvement in symptoms. Biventricular device upgrade was considered, which was not pursued as the patient’s LVEF was normal.

The patient’s symptoms, which limited his daily activities, persisted without progression for the next 2 months; he then presented to the electrophysiology clinic for follow-up. During the clinic visit, his vital signs were normal and the physical examination was unremarkable, with no signs of pulmonary edema or jugular venous distension (however, evaluation of the vein was limited due to obesity).

**Figure 3:**
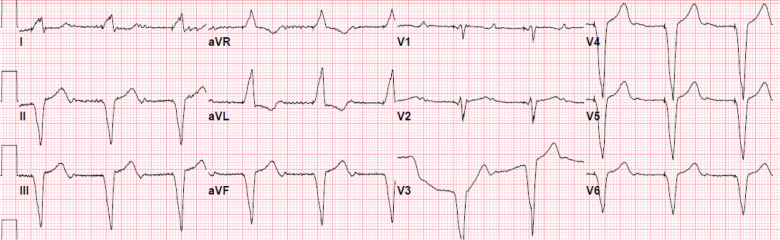
Electrocardiogram obtained during the clinic visit. Ventricular pacing was at 60 ppm. P-waves are seen 500 ms after each ventricular pacing spike.

**Figure 4:**
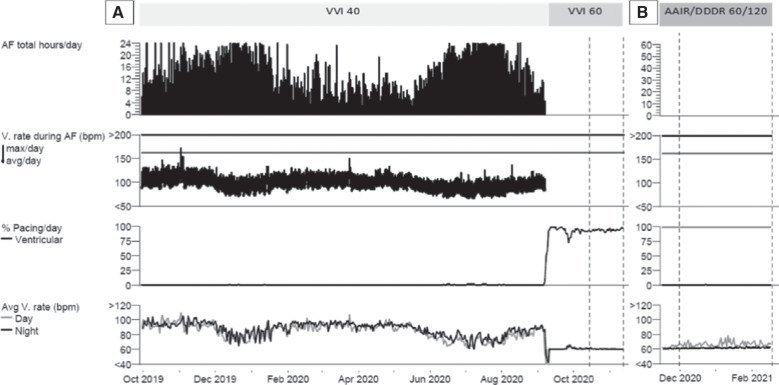
Composite device follow-up data. **A:** From October 2019 to September 2020: a single-chamber implantable cardioverter-defibrillator (ICD) was set at VVI 40 ppm. From September 2020 to November 2020, the same single-chamber ICD set at VVI 60 ppm. **B:** In November 2020, the device was upgraded to a dual-chamber ICD, was set at AAIR/DDDR 60/120 ppm.

The presentation of sudden-onset persistent dyspnea with a stable course suggested the occurrence of an event tht has remained uncorrected for months. Common cardiovascular, pulmonary, and extracardiac culprits have already been ruled out. Due to the prominent finding of the sudden onset and markedly lower heart rate when the patient’s symptoms started, arrhythmogenic causes were considered. Ventricular pacing may lead to ventricular dyssynchrony and cardiomyopathy; however, his LVEF actually improved. Issues with atrioventricular synchrony were then considered. An ECG was obtained **(Figure 3)**, and the ICD was interrogated **(Figure 4A)**. The ECG showed ventricular pacing with 1:1 retrograde conduction and long ventriculoatrial delay with negative/biphasic P-waves in the inferior leads occurring 500 ms after the ventricular pacing spike. Similar retrograde atrial activation was already present, as shown in **Figure 2**, with P-waves buried in the T-waves as the retrograde conduction interval was shorter, around 450 ms. In contrast, the ECG obtained after the ICD implant did not show retrograde P-waves after the ventricular paced beat, as the patient was in AF **(Figure 1)**.

The sudden onset of symptoms is consistent with pacemaker (PM) syndrome: the patient had spontaneous conversion of long-standing persistent AF, and VVI pacing was initiated due to sinus bradycardia, leading to 1:1 retrograde conduction. The AF detection algorithm underestimated the burden as it is based on single-chamber data. The ventricular pacing remained close to 100% for the next 2 months at 60 ppm **(Figure 4A)**.

**Figure 5:**
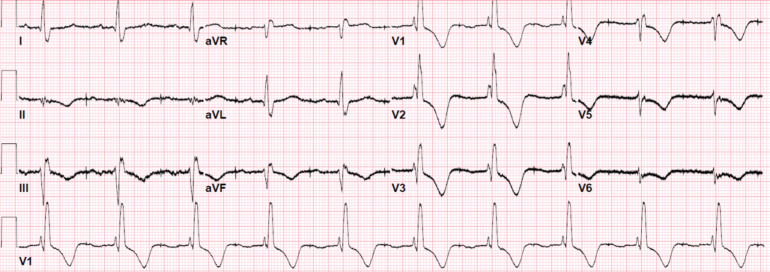
Electrocardiogram obtained after the dual-chamber device upgrade. Atrial pacing was set at 60 ppm with prolonged atrioventricular conduction, right bundle branch block, and left anterior fascicular block.

The ICD was later upgraded to a dual-chamber system set to AAIR/DDDR at 60 ppm with an upper sensor rate of 120 ppm. Post-implant ECG showed atrial pacing at 60 bpm, with prolonged atrioventricular conduction and unchanged intraventricular conduction delay **(Figure 5)**. The patient’s shortness of breath promptly improved after the device upgrade. Amiodarone and metoprolol succinate were continued for rhythm control of AF and ventricular tachycardia, and anticoagulation was also continued. After the device upgrade, no recurrence of AF was noted; atrial pacing remained at 100%, while ventricular pacing decreased to <1% **(Figure 4B)**.

Informed consent was obtained for all procedures. No ethical approval was needed.

## Discussion

PM syndrome is a known complication of single-chamber ventricular pacing. Atrial contraction against closed atrioventricular valves decreases the preload for the next cardiac cycle and increases venous pressure. Symptoms include shortness of breath, dyspnea on exertion, hypotension, and presyncope or even syncope.^1^ The cause may be difficult to identify if the loss of atrioventricular synchrony is intermittent. Physical signs include elevated jugular venous pressure/venous pulsation, cannon A-waves, and sometimes pulmonary edema. The incidence of PM syndrome among patients with single-chamber devices was estimated to be around 25% in early PM trials.^2^ However, the emergence of transfemoral single-chamber PM implants increased the number of patients who may be at risk. Clinically, this does not seem to be a common occurrence, despite the fact that around 30% of patients receiving a VVI device for sinus node dysfunction fall into this group.^3^ Changing the ventricular pacing rate may not resolve this issue if retrograde conduction is preserved; an upgrade to a dual-chamber system helps to restore synchrony.

The upgrade led to 3 benefits in our patient, which were restoration of atrioventricular synchrony/elimination of PM syndrome, rate-responsive atrial pacing, and a slightly narrower QRS duration (underlying bifascicular block compared to ventricular pacing). We hypothesize that the elimination of PM syndrome led to the improvement of symptoms, as the average heart rate remained close to the base heart rate and the change in the QRS duration was minimal—that is, the symptoms promptly resolved once atrial pacing was initiated, as this eliminated atrial contraction against the closed mitral and tricuspid valves (caused by ventricular pacing with intact retrograde conduction).

Spontaneous or pharmacological cardioversion of long-standing persistent AF is rare, and rhythm control is difficult in these patients.^4^ Based on the current pacing guidelines, implant of an atrial lead is contraindicated in persistent AF if rhythm control is not pursued.^5^ Conversion of long-standing persistent AF may occur in rare cases: our patient had good cardiac reverse remodeling after valve surgery and revascularization. Post-conversion, bradycardia may be observed due to sinus node dysfunction; in our patient, this persisted, and he required 100% atrial pacing after the upgrade. Amiodarone, used to suppress ventricular tachycardia, may have contributed to the conversion; this patient took amiodarone for 12 months prior to the AF termination. Conversely, this phenomenon has been observed in patients with advanced atrial remodeling, such as rheumatic mitral heart valve disease, with resulting slow sinus or junctional rhythm.^6^ The mechanism is probably related to a loss of atrial myocardium to maintain AF.

## Conclusion

Spontaneous termination of long-standing persistent AF is uncommon but should be considered in patients in whom ventricular pacing suddenly elicits symptoms suggestive of PM syndrome. A 12-lead ECG should be evaluated carefully for signs of retrograde atrial activation. Device reprogramming or upgrade should be considered when PM syndrome due to ventricular pacing is encountered.
